# Regulation of dual specificity phosphatases in breast cancer during initial treatment with Herceptin: a Boolean model analysis

**DOI:** 10.1186/s12918-018-0534-5

**Published:** 2018-04-11

**Authors:** Petronela Buiga, Ari Elson, Lydia Tabernero, Jean-Marc Schwartz

**Affiliations:** 1 Department of Molecular Genetics, The Weizmann Institute of Science, Rehovot, Israel; 2 School of Biological Sciences, Faculty of Biology, Medicine and Health, University of Manchester, Manchester, UK

**Keywords:** Boolean model, Herceptin, Breast cancer, DUSPs

## Abstract

**Background:**

25% of breast cancer patients suffer from aggressive HER2-positive tumours that are characterised by overexpression of the HER2 protein or by its increased tyrosine kinase activity. Herceptin is a major drug used to treat HER2 positive breast cancer. Understanding the molecular events that occur when breast cancer cells are exposed to Herceptin is therefore of significant importance. Dual specificity phosphatases (DUSPs) are central regulators of cell signalling that function downstream of HER2, but their role in the cellular response to Herceptin is mostly unknown. This study aims to model the initial effects of Herceptin exposure on DUSPs in HER2-positive breast cancer cells using Boolean modelling.

**Results:**

We experimentally measured expression time courses of 21 different *DUSP*s between 0 and 24 h following Herceptin treatment of human MDA-MB-453 HER2-positive breast cancer cells. We clustered these time courses into patterns of similar dynamics over time. In parallel, we built a series of Boolean models representing the known regulatory mechanisms of DUSPs and then demonstrated that the dynamics predicted by the models is in agreement with the experimental data. Furthermore, we used the models to predict regulatory mechanisms of DUSPs, where these mechanisms were partially known.

**Conclusions:**

Boolean modelling is a powerful technique to investigate and understand signalling pathways. We obtained an understanding of different regulatory pathways in breast cancer and new insights on how these signalling pathways are activated. This method can be generalized to other drugs and longer time courses to better understand how resistance to drugs develops in cancer cells over time.

**Electronic supplementary material:**

The online version of this article (10.1186/s12918-018-0534-5) contains supplementary material, which is available to authorized users.

## Background

Breast cancer represents a major public health issue, affecting one in eight women in the Western world [1]. Approximately 25% of human breast cancer cases are HER2-positive, due to overexpression of the HER2 protein or amplification of the *HER2* gene, or due to activating mutations that increase HER2 activity. HER2-positive tumours are associated with poor prognosis [2, 3]. HER2 is a 185 kDa protein that belongs to the family of tyrosine kinase epidermal growth factor receptors, and is also known as Neu or ErbB2. HER2 has no known ligand and is activated by dimerization or by incorporation into heterodimers with its related family members HER1, HER3 or HER4 [4]. Increased membrane receptor concentration of HER2 in HER2-overexpressing cells can also activate HER2 by ligand-independent homo-dimerization, leading to activation of the HER2 signalling pathway [5]. Activated HER2 auto-phosphorylates and associates with other molecules, activating several signalling pathways and mediators, such as the Mitogen-Activated Protein Kinases (MAPKs), which promote cell growth, proliferation, and survival [6].

A major drug for treating HER2-positive breast cancer is Herceptin, a humanized monoclonal antibody that binds the extracellular Domain IV of HER2. Herceptin reduces HER2 dimerization leading to, among other effects, inhibition of MAPK signalling [6]. Herceptin has high efficacy in patients with HER2-positive breast cancer, but resistance to its effects often develops and poses a significant therapeutic challenge [7, 8].

MAPKs, such as ERK1, ERK2, p38 and JNK, are major regulators of HER2 signalling and of other signalling events that take place in cancer cells. MAPKs are activated by phosphorylation of specific threonine and tyrosine residues present in a TXY motif by upstream kinases. Dephosphorylation of either or both the threonine and tyrosine residues of this motif downregulates MAPK activity. Dephosphorylation of both residues is carried out by specialized enzymes, the dual-specificity phosphatases (DUSPs) that are part of the larger tyrosine phosphatase superfamily [9]. DUSPs comprise a subfamily of 10 MAPK phosphatases (MKPs) that are key players in inactivating MAPKs, leading to down-regulation of MAPK signalling [10, 11]. A second subfamily of DUSPs, the atypical DUSPs, are also involved in regulating the same pathways [12]. Each DUSP has different subcellular localization and substrate specificity [13]. DUSPs are of particular interest in the context of cancer, since they play putative roles in tumour progression and in resistance to therapy [14]. Deeper understanding of the mechanisms that regulate DUSP expression in HER2 signalling is crucial to clarify their contribution in cancer promotion and in development of resistance of Herceptin. A good experimental model to study HER2-positive breast cancer is MDA-MB-453, a human breast cancer cell line that overexpresses endogenous HER2 protein [15, 16].

One of the most useful methods to predict the outcome of signalling pathways is a systems biology approach through logical modelling [17]. Logical models such as Boolean models are powerful tools to represent dynamic molecular processes in complex cellular systems, where construction of detailed kinetic models would be prohibitively expensive and time-consuming, or where complete knowledge of molecular interactions or quantitative details of biological processes is lacking [18]. In a Boolean model, nodes represent biological components of interest and are connected by edges describing the nature of the interaction occurring between them. The activity of biological components is represented by discrete values (states), generally 0 (OFF, inactive) or 1 (ON, active), and actions are represented by activation (positive regulation) or inhibition (negative regulation) [18].

Several studies of qualitative and quantitative modelling related to HER signalling have been performed. Chen et al. presented a quantitative model of HER signalling during the immediate-early phase of ligand-stimulated cell signalling [19]. Samaga and colleagues built a comprehensive logical model of the signalling network of EGFR in liver cancer, which was based on the comprehensive EGFR network map published by Oda and colleagues [20, 21]. Von der Hyde et al.*,* constructed a Boolean model of HER2 signalling to identify individual drug response patterns and their resistance in HER2-positive breast cancer [22]. However, none of these studies addressed DUSPs specifically, leaving a major gap in our understanding of how Herceptin affects these important regulators of cell signalling.

Our work analyses Herceptin action on DUSP expression in HER2-positive breast cancer cells, in order to investigate and in some cases predict regulatory mechanisms for *DUSP*s in this context. The current study examines DUSPs during the initial phase of Herceptin treatment, in which some changes in gene expression occur, but which is still too early for compensatory mutations to arise or to be selected for. Initially, *DUSP* mRNA expression in HER2-positive breast cancer cells was determined experimentally during the period between 0 to 24 h following cell exposure to Herceptin. In parallel, we constructed a series of models that collectively accounted for the response of each *DUSP* to the drug and for the variations in responses among DUSPs, and enabled prediction of new regulatory mechanisms that were unknown.

## Methods

### Cell line

The human breast cancer cell line MDA-MB-453 (American Type Culture Collection, Manassas, VA, USA) was cultured in RPMI 1640 medium (Gibco, Carlsbad, CA, USA) containing 50 U/ml penicillin, 50 mg/ml streptomycin and 10% heat-inactivated foetal bovine serum (FBS - Gibco) at 37 °C in an atmosphere of 5% CO_2_.

### Herceptin treatment

Cells were plated in 60 mm plates in duplicate for each time point, and cultured for two days until they reached 75% confluency. Cells were then treated with 50 μM Herceptin (Roche, Switzerland) continuously for 2, 4, 12 or 24 h and then collected. Plates prepared and grown in parallel but not exposed to Herceptin were used as time 0. Total RNA was extracted from cells using the RNeasy Mini Kit (QIAGEN, Germany) and was treated with DNase I at room temperature for 15 min. One microgram of total cellular RNA was reverse transcribed in a final volume of 20 μl using the qScript cDNA synthesis kit (Quanta Biosciences, Beverly, MA, USA) according to the manufacturer’s protocol. 0.5 μl (25 ng) cDNA was used to perform quantitative PCR (RT-qPCR) using KAPA SYBR Fast qPCR Master Mix (2X) ABI Prism (Kapa Biosystems, Wilmington, MA, USA) and target-specific forward and reverse primers (Additional file 1) in an AB StepOnePlus instrument (Applied Biosystems, Foster City, CA, USA).

The amplification conditions consisted of an initial activation step of 95 °C for 20 s, followed by 40 cycles of 95 °C for 3 s and 60 °C for 30 s, and ending with one run of 95 °C for 15 s, 60 °C for 60 s and 95 °C for 15 s.

Standard curves were used to assess primer efficiency, and average change in threshold cycle (ΔCT) values were determined for each of the samples relative to endogenous Actinb and GAPDH (internal control) levels using the ΔΔCT method, where the fold change of target gene expression is determined relative to a reference sample, normalized to the reference genes (Actinb and GAPDH) [23]. Experiments were performed in triplicates to determine mean along with standard error, having two biological repeats. DUSP-specific forward and reverse primers were designed using the Primer3 software [24, 25].

Levels of Actinb and GAPDH mRNA transcripts measured as described above were used to normalize the amount of input RNA amplified in each reaction. Each sample was amplified in duplicate and the resultant mean values were used for analysis. Student T-test statistical analysis was performed in GraphPad Prism version 7.0a for Mac OS X (GraphPad Software, La Jolla California, USA).

### Correlations of *DUSP* expression

*DUSP*s time courses were further analysed by computing Pearson correlation coefficients between all time series; the log_10_ of raw intensity values was taken, then correlations were computed using the *corr* function in Python 2.7. Since we wanted clusters to be composed of only positively correlated time courses, we set all negative correlations to zero then computed clusters using the *clustermap* function from the *seaborn* library, with the default average linkage method; clusters were plotted using *matplotlib*.

### Construction of Boolean models

Boolean models were manually constructed following an extensive literature review. Previously reported network maps of HER signalling were mined to find the essential proteins and interactions for HER2 downstream signalling. Relevant proteins and interactions found to be involved in downstream signalling of MAPK - ERK as described by Chen et al., and other components of MAPK downstream signalling, JNK and p38, in models developed by Oda et al. and Samaga et al., were included. All the selected interactions have been checked for cellular context and verified using multiple literature sources [19–21].

In normal cells, the basal level of the downstream MAP kinases (ERK, JNK and p38) leads to proliferation; increased activity of both JNK and p38 leads to apoptosis and inhibition of cellular growth and proliferation [26–29]. However, cancer cells often do not undergo apoptosis because of the inhibitory action of DUSPs on both JNK and p38. Detailed interactions of DUSPs with their cognate kinases were not included in any of the previously reported maps or models. These were added to our models, based on literature reports of substrate specificity [12, 13] and the necessity to investigate their contribution.

Graphical maps of all the models were created using the yWorks yEd graph editor (version 3.14.4). All the interacting components of the model were represented as nodes (rectangular boxes), while the interactions were represented as edges (sharp arrow for activation and blunt arrow for inhibition). Circles represent the AND function which combines several interactions. Model construction and time course simulation of MAPK signalling pathways in response to Herceptin treatment was done using the BooleanNet toolkit under Python 2.7 [30]. Both synchronous and asynchronous simulations were performed. In synchronous updating rules, all variables are updated at the same time, while in asynchronous simulations a single random variable is updated at a time. The asynchronous simulations confirmed the results obtained from the synchronous simulations, hence we only present the results of the latter. Heatmaps were generated and exported using the Python tool matplotlib [31].

Herceptin was selected to be a user defined input, while Survival was chosen as a readout. Each node in the model can be either in an active state (ON) or in an inactive state (OFF), referring to 1 and 0 respectively in a binary domain *n* ∈ ℕ; *t* ∈ ℤ+: *n*(*t*) = 0 or 1. In this model interactions were represented as either activation or inhibition, combined using AND, OR and NOT Boolean functions, which are sufficient to represent any logical relationship. All of these reactions were considered to have equal time scale *t* = 1. A set of initial conditions were assumed considering at least one node to be active, then other nodes either become inactive (1 ➔ 0), active (0 ➔ 1) or maintain their state (0 ➔ 0 or 1 ➔ 1) after each time increment. These changes are dependent on the upstream nodes and interaction conditions. Where difficulties arose in determining the use of AND or OR functions, this was solved by reviewing the specific literature. The AND function was used when there was an explicit action of two or more proteins acting collectively on a node, otherwise the OR function was applied [32].

## Results

### *DUSP* expression measured by qPCR

**Fig. 1:**
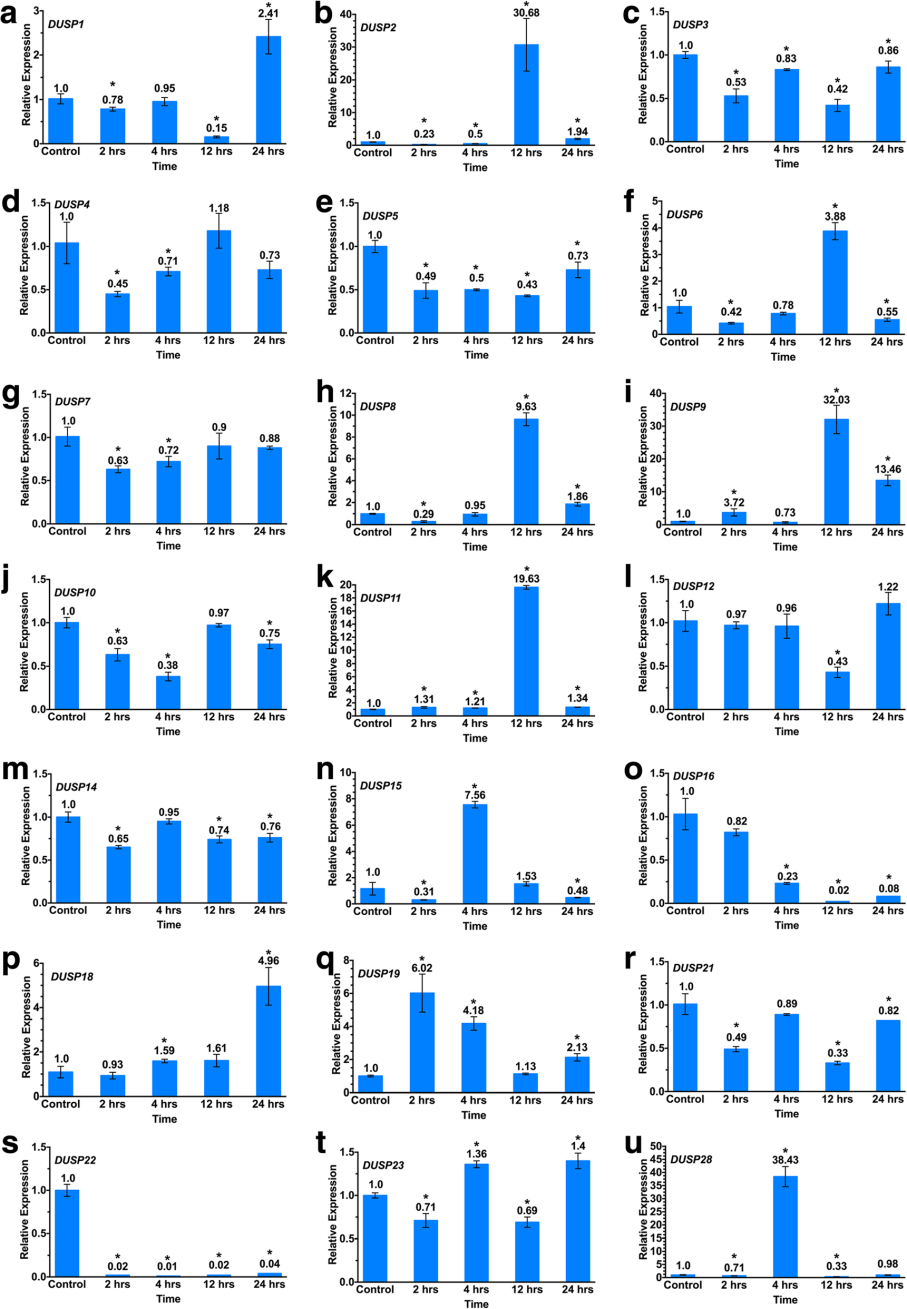
*DUSP* mRNA expression measured by RT-qPCR. (**a**-**u**) *DUSP* expression measured by RT-qPCR, comparing expression of the indicated *DUSP*s in control (non-treated, time 0) samples with cells at 2, 4, 12 and 24 h following exposure to Herceptin. Bars represent mean ± SE. Two biological repeats (each in triplicate) were performed. An asterisk indicates a statistically-significant difference of *p* < 0.05 from the value at t = 0

We measured gene expression of 21 *DUSP*s (10 MKPs and 11 atypical *DUSP*s) by RT-qPCR in the human HER2-positive MDA-MB-453 breast cancer cell line at 0, 2, 4, 12 and 24 h following Herceptin exposure. Data from time point 0 (non-treated samples) was the reference against which expression levels at the other time points were compared for each *DUSP*. We limited our analysis to 24 h in order to focus on the initial response in *DUSP* gene expression and cell signalling to Herceptin. RT-qPCR expression levels of the 21 selected *DUSP*s are shown in Fig. 1(a-u).

Expression of individual *DUSP*s was variable among the different time points following Herceptin treatment. Herceptin treatment led to a decrease in expression of most *DUSPs*: expression of *DUSP*s 3, 5, 7, 10, 14, 16, 21 and 22 was decreased during the entire exposure to Herceptin, while expression of *DUSP*s 1 and 12 was decreased until 12 h. Some of the *DUSP*s showed no clear trend or no strong change, while other *DUSP*s decreased at the beginning, but rose strongly at later time points (e.g. *DUSP*9). Expression of a number of *DUSP*s increased immediately, but subsequently decreased: *DUSP*19 increased up to 2 h and decreased later. *DUSP*s 2, 6 and 8 showed a peak at 12 h, *DUSP*28 and 15 showed a peak at 4 h. *DUSP*22 expression was abolished over the entire time series, while in *DUSP*16 expression showed a gradual decrease. In contrast, *DUSP*18 showed a gradual increase in expression throughout the entire time course of this study.

### Correlation of *DUSP* expression and clustering

**Fig. 2:**
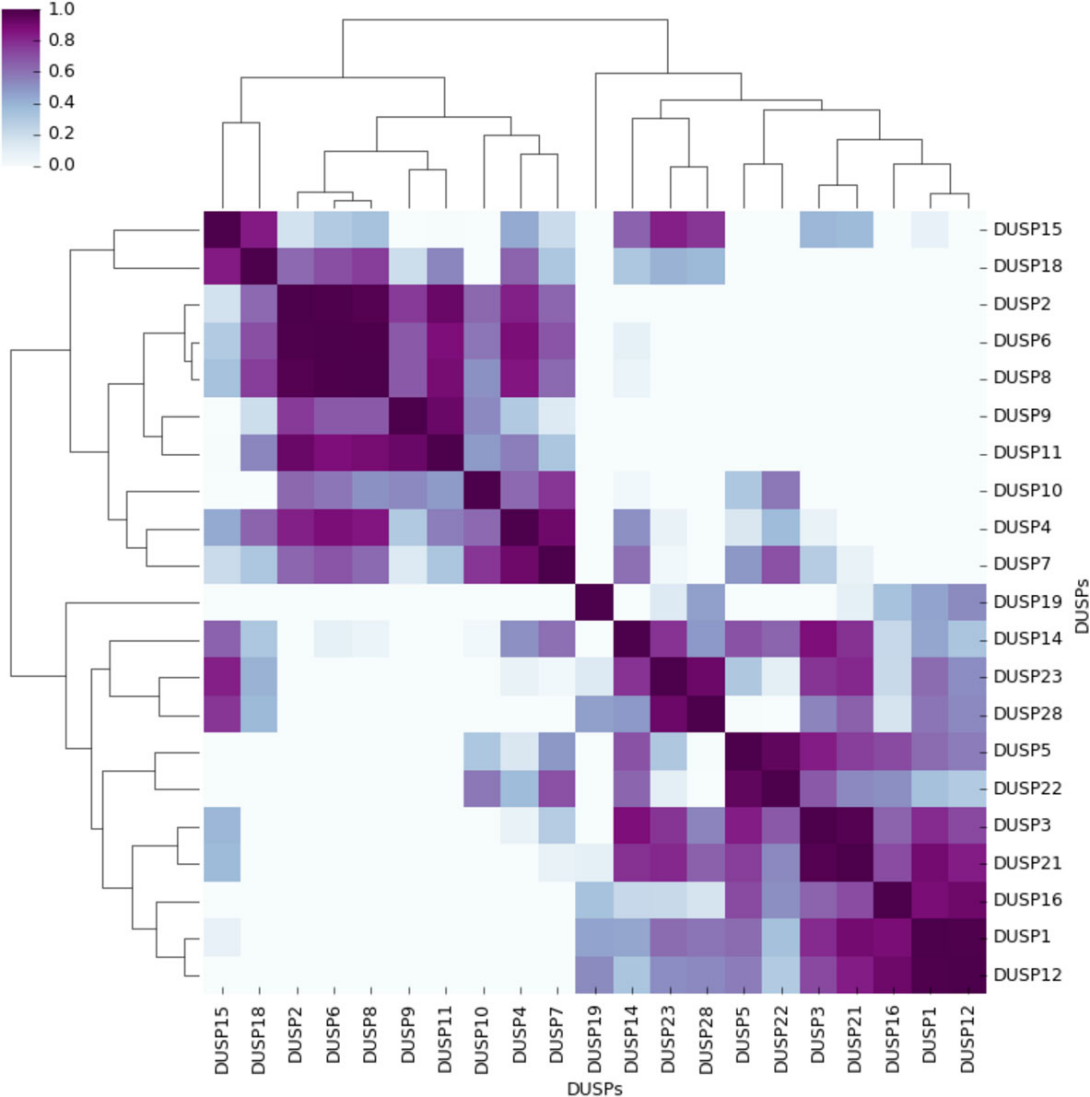
Clustering of *DUSP* expression time courses. Clustering of *DUSP* expression time courses between 0 and 24 h after exposure to Herceptin. Colours represent the Pearson correlation coefficient between each pair of *DUSP*s, with negative values clipped to zero

In order to characterise these different expression patterns in a more rigorous manner, we calculated correlations between expression time courses of all individual *DUSP*s. Figure 2 shows an overview of matching *DUSP* expression patterns, which revealed seven clusters that were selected for further modelling and analysis.

From the correlation matrix and visual inspection of the expression time courses we defined the following clusters: cluster 1 is composed of *DUSP*s 2, 6 and 8; cluster 2 includes *DUSP*s 9 and 11; *DUSP*s 4 and 7 belong to cluster 3; cluster 4 consists of *DUSPs* 23 and 28; *DUSPs* 5 and 22 are part of cluster 5; *DUSPs* 3 and 21 belong to cluster 6, while cluster 7 includes *DUSPs* 1 and 12. There were six remaining *DUSPs* which did not fit into any cluster, these are: *DUSPs* 10, 14, 15, 16, 18 and 19.

**Fig. 3:**
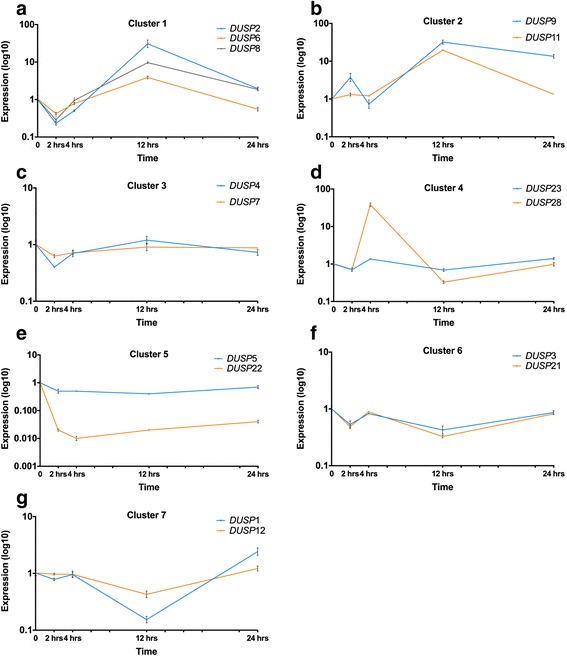
Time series of *DUSP* expression exposed to Herceptin grouped by selected clusters. The horizontal axis represents time, the vertical axis represents measured expression expressed in logarithmic scale. Expression levels were normalised with respect to the control (1). Data represents mean ± SE (two biological repeats, each in triplicate) as noted in Fig. 1. **a** Cluster 1; **b** Cluster 2; **c** Cluster 3; **d** Cluster 4; **e** Cluster 5; **f** Cluster 6; **g** Cluster 7

The distinct expression patterns over time for each cluster are shown in Fig. 3, where expression of all *DUSP*s in a given cluster is plotted on logarithmic scale. Generally, an immediate decrease in *DUSP* expression can be seen in the clusters. For cluster 1, the immediate decrease is followed by a sharp increase after 2 h then an abrupt reduction after 12 h. Cluster 2 shows little change for the first 4 h, followed by a marked increase until 12 h, ending with a slight decrease until 24 h. In cluster 3, an initial reduction for the first 4 h is followed by an increase in *DUSP* expression until 12 h. Cluster 4 shows an immediate decrease then abrupt increase at 4 h, followed by a sharp decrease until 12 h, ending with an increase until 24 h. In cluster 5 the expression shows a moderate oscillating pattern, the expression varies within a small range. In cluster 6 a reduction in *DUSP* expression is observed until 12 h followed by an increase until 24 h. Finally, cluster 7 shows an overall pattern of a slight increase followed by a sharp decrease.

### Boolean modelling of the selected clusters

**Fig. 4:**
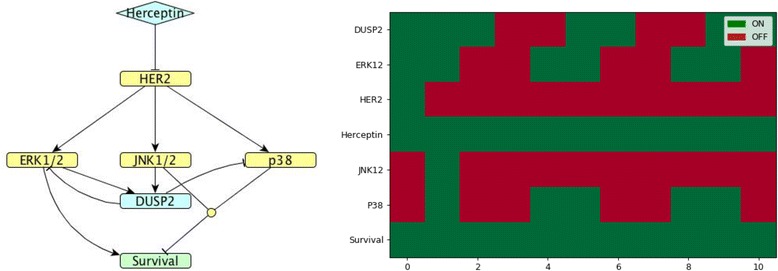
*DUSP*2 model. (Left). Network map of *DUSP*2 regulation (cluster 1) in MDA-MB-453 HER2-positive breast cancer cells exposed to Herceptin. (Right). Corresponding Boolean time series represented as a heatmap showing gene expression. Green and red indicate nodes in the ON and OFF states, respectively. Note that time units in Boolean simulations are arbitrary and do not necessarily match the 24 h period of the study, but the overall expression pattern can be compared to the overall Boolean time series

**Fig. 5:**
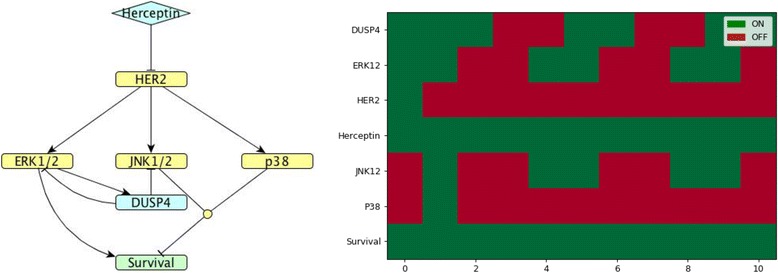
*DUSP*4 model. (Left). Network map of *DUSP*4 (cluster 3) regulation in MDA-MB-453 HER2-positive breast cancer cells exposed to Herceptin. (Right). Corresponding Boolean time series represented as a heatmap showing gene expression as described in Fig. 4

**Fig. 6:**
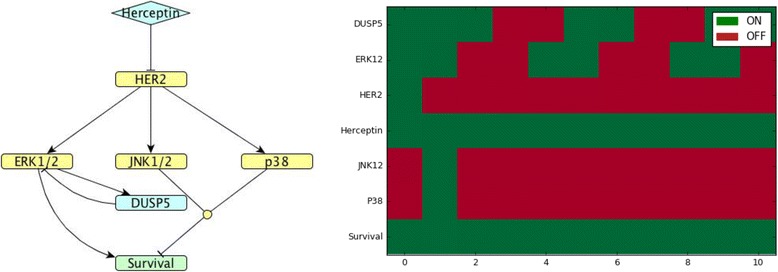
*DUSP*5 model. (Left). Network map of *DUSP*5 (cluster 5) regulation in MDA-MB-453 HER2-positive breast cancer cells exposed to Herceptin. (Right). Corresponding Boolean time series represented as a heatmap showing gene expression as described in Fig. 4

**Fig. 7:**
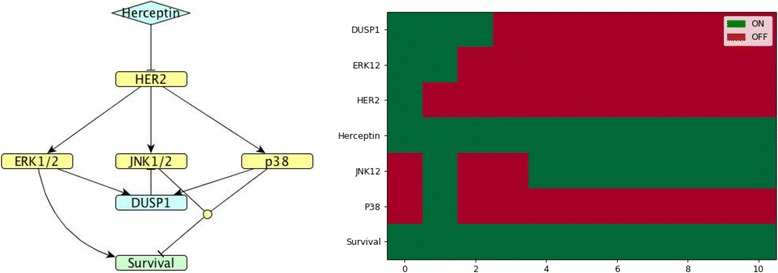
*DUSP*1 model. (Left). Network map of *DUSP*1 (cluster 7) regulation in MDA-MB-453 HER2-positive breast cancer cells exposed to Herceptin. (Right). Corresponding Boolean time series represented as a heatmap showing gene expression as described in Fig. 4

In order to interpret the biological findings, a series of models were created to simulate and represent the possible combinations of *DUSP* regulation (including induction of expression and substrate specificity). Each analysed cluster has been matched with a model. Figures 4-7 show the model and simulation results for one representative *DUSP* from each cluster for which regulatory mechanisms were known: *DUSP*2 for cluster 1, *DUSP*4 for cluster 3, *DUSP*5 for cluster 5 and *DUSP*1 for cluster 7.

The representative of cluster 1, DUSP2, is induced by ERK1/2 and JNK1/2; it inhibits ERK1/2 and p38 [13]. When exposed to Herceptin over time, *DUSP*2 shows an oscillating pattern in the Boolean model that agrees with the quantified gene expression data by RT-qPCR (see Figs. 3a and 4).

The regulation of DUSP4, the chosen representative of cluster 3, is known: it is induced by ERK1/2 and it inhibits ERK1/2 and JNK1/2 [13]. When exposed to Herceptin over time, *DUSP4* shows oscillating expression in the Boolean model that agrees with the quantified gene expression data by RT-PCR (see Figs. 3c and 5).

The representative of cluster 5, DUSP5, is induced by ERK1/2 and it inhibits ERK1/2 [13]. When exposed to Herceptin over time, DUSP5 shows oscillating behaviour in the Boolean model that agrees with the quantified gene expression data by RT-PCR (see Figs. 3e and 6).

DUSP1, the representative for cluster 7, is induced by ERK1/2 and p38, and inhibits JNK1/2 [13]. When exposed to Herceptin, Boolean modelling indicates that *DUSP1* expression is initially stable and then decreases, in accordance with the quantified gene expression data by RT-PCR up to 12 h (see Fig. 3g and 7).

**Fig. 8:**
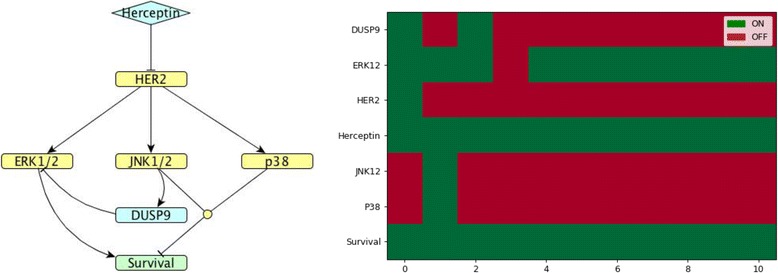
*DUSP*9 model. (Left). Network map of *DUSP*9 (cluster 2) regulation in MDA-MB-453 HER2-positive breast cancer cells exposed to Herceptin. (Right). Corresponding Boolean time series represented as a heatmap showing gene expression as described in Fig. 4

**Fig. 9:**
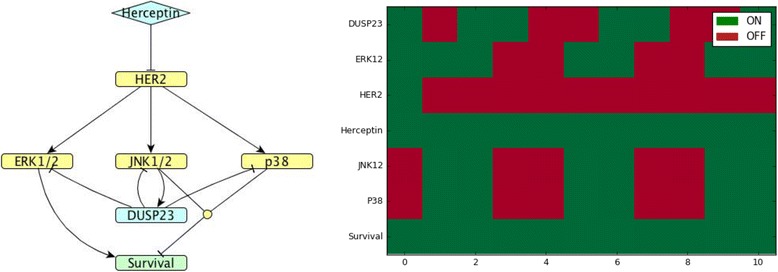
*DUSP*23 model. (Left) Network map of *DUSP23* (cluster 4) regulation in MDA-MB-453 HER2-positive breast cancer cells exposed to Herceptin. (Right). Corresponding Boolean time series represented as a heatmap showing gene expression as described in Fig. 4

**Fig. 10:**
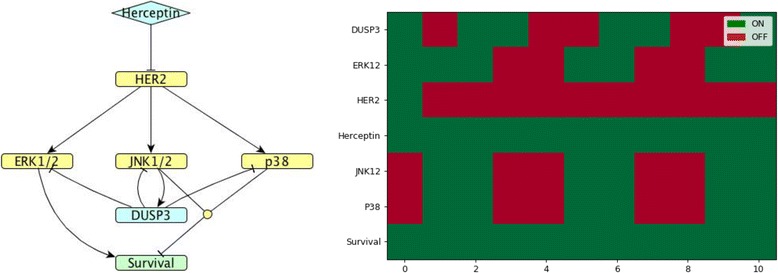
*DUSP*3 model. (Left). Network map of *DUSP*3 (cluster 6) regulation in MDA-MB-453 HER2-positive breast cancer cells exposed to Herceptin. (Right). Corresponding Boolean time series represented as a heatmap showing gene expression as described in Fig. 4

Figures 8-10 show representative models matching expression trends with their corresponding predicted heatmaps for *DUSP*s whose regulation is only partially known. Each model and heatmap shows one representative *DUSP* per cluster: *DUSP*9 (cluster 2), *DUSP*23 (cluster 4) and *DUSP*3 (cluster 6). For all these *DUSP*s, it is currently unknown by which MAPKs they are induced.

In cluster 2, *DUSP*9 was chosen as a representative and its regulation is partially known: its substrate is ERK, but it is unknown by which of the MAP kinases it is induced. Therefore, we simulated the possible combinations of all regulators and we found that the only solution that showed a convincing agreement between Boolean model simulation and experimental time course was the one where *DUSP*9 is induced by JNK. The agreement between Boolean model (Fig. 8) and *DUSP*9 expression time course enables us to predict that *DUSP9* might be regulated by JNK. Since the two MAP kinases JNK and p38 are symmetrical in the model, the same outcome can be achieved if *DUSP*9 is induced by p38.

In cluster 4, *DUSP*23 is the smallest active phosphatase, whose regulation is only partially known: its substrates are ERK [33], JNK1/2 and p38 [34], but it is unknown by which of the MAP kinases it is induced. The Boolean model shown in Fig. 9 agrees with the experimental time course and enables us to predict that *DUSP*23 might be regulated by JNK1/2. Since the two MAP kinases JNK and p38 are symmetrical in the model, the same outcome can be achieved if *DUSP*23 is induced by p38.

In cluster 6, *DUSP*3 is an atypical phosphatase that has a similar catalytic domain as the typical MKPs. Therefore, *DUSP*3 was suggested to dephosphorylate MAP kinases in the same manner as MKPs [12]: JNK, ERK [35] and also p38 [36]. The Boolean simulation shown in Fig. 10 agrees with the experimental time course, enabling us to predict that *DUSP3* might be regulated by JNK1/2.

**Fig. 11:**
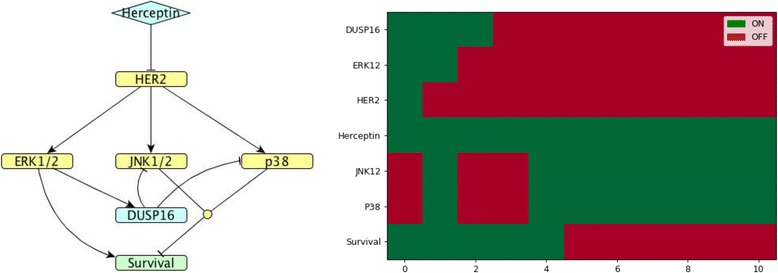
*DUSP*16 model. (Left). Network map of *DUSP*16 regulation in MDA-MB-453 HER2-positive breast cancer cells exposed to Herceptin. (Right). Corresponding Boolean time series represented as a heatmap showing gene expression as described in Fig. 4

*DUSP*16 has an inhibitory action on JNK and p38 [13], but it is unknown by which kinase it is induced. The model presented in Fig. 11 is in agreement with the measured *DUSP*16 expression time course, therefore we can predict that *DUSP*16 might be induced by ERK. It is worth noting that all models presented above show survival to be ON, but in the case of the *DUSP*16 model, survival switches to OFF after some time.

## Discussion

Previous studies of Herceptin treatment in HER2 positive breast cancer and development of resistance to Herceptin focused on its effects on various signalling pathways and on cell survival [22, 37–39]. Györffy and colleagues correlated Herceptin resistant tumours from patients with DUSPs involved in development of Herceptin resistance in HER2 positive breast cancer, and suggested that DUSP4 is involved in this process [40]. Nonetheless, there are significant gaps in our understanding of whether and how DUSPs participate in development of Herceptin resistance in breast tumours.

Here we presented a comprehensive analysis of the early-stage responses of 21 selected *DUSP*s to Herceptin treatment in human breast cancer cells. The cell line MDA-MB-453 is reported in the literature in some cases as being Herceptin sensitive [41–43] and in others as Herceptin resistant [44–46]. Our study focuses on short-term effects of exposure to Herceptin in order to examine the initial response of HER2-expressing tumour cells to Herceptin. We expect that this response is based on the pre-existing cell signalling pathways – possibly with some rapid re-configuration induced by Herceptin – and would shed light on the structure of these pathways. In contrast, the time frame of this study effectively prevents mutations in genes from affecting the cellular response. *DUSP*s 1, 3, 4, 7, 10, 12, and 21 showed a decrease in expression compared to the control, non-treated group during the entire time series, while the other *DUSP*s showed a pattern of increasing expression over time. Herceptin reduces mitogenic and survival signals from HER2. It is then possible that the initial decrease in *DUSP* expression aims to counter this effect by removing DUSPs that down-regulate MAPK activity and thus also counter mitogenic and survival signalling. At later times additional roles of *DUSP*s become evident, and their expression patterns diverge. For example, at 24 h expression of *DUSP*s 1, 12, 23, 28, 3, 21 increases, possibly indicating the beginning of emerging resistance. Alternatively, these *DUSP*s may perform additional roles upon which cell survival depends and which limit the length of time the cells can withstand their reduced expression.

Uniquely for *DUSP*16, whose expression is decreased dramatically following treatment with Herceptin, the model shows that Survival switches OFF after some time. Although cells are not dying in the time frame of 24 h, reduced expression of phosphatase might play an important role in driving apoptosis of cells treated with Herceptin, and thus prevent resistance to the drug from arising. Additional experimental and modelling studies are therefore required to understand cell behaviour at later time points.

Previous studies have utilized Boolean modelling to understand cellular responses in cancer. Fumia and Martins constructed and described theoretical models which included the main signalling pathways in cancer in order to interpret mutational events and cancer cell response [47]; Grieco et al. performed in silico simulations to understand the role of deregulations in MAPK signalling on cell fate decision in cancer [48]. Von der Heyde et al. used reverse and forward engineering techniques in order to reveal individual mechanisms of drug response or resistance in breast cancer [22]. Sahin et al. constructed a literature-based Boolean model to model the regulation of pRB through ERBB-receptor signalling in native Herceptin resistant cell lines. These authors integrated the experimental data into their model and overcame discrepancies with additional experimental validation [49]. Our approach is distinct in that we combine pre-existing biological knowledge about *DUSP* regulation to construct mechanistic Boolean models, together with independent experimental analysis to challenge and validate these models. The models presented here were not trained to fit experimental data but we showed that the predicted dynamics of *DUSP* expression is in accordance with experimentally observed time courses, which gives stronger confidence that the models are mechanistically correct. Moreover, this approach allowed us to predict new regulatory mechanisms for DUSPs with partially known regulation, such as DUSPs 9, 23, 3 and 16. In the future, this method can be extended by measuring and simulating longer time series, as well as by including additional signalling pathways in order to better understand the dynamics of development of resistance to Herceptin in breast cancer.

## Additional file

Additional file 1: List of primers used for qPCR analyses of the expression of the *DUSP* mRNAs examined in this study. Description: All sequences are of human origin except for beta actin, which is from mouse. (DOCX 198 kb)
