# Supplementary material for: Regulation of dual specificity phosphatases in breast cancer during initial treatment with Herceptin: a Boolean model analysis
Source: BMC Syst Biol. 2018 Apr 11;12(Suppl 1):11. doi: 10.1186/s12918-018-0534-5 (PMC5907139; doi:10.1186/s12918-018-0534-5)
Supplement: Supplementary file 1 — List of primers used for qPCR analyses of the expression of the DUSP mRNAs examined in this study. Description: All sequences are of human origin except for beta actin, which is from mouse. (DOCX 198 kb) [file 12918_2018_534_MOESM1_ESM.docx]

**Additional file 1**

**List of primers used for qPCR analyses of the expression of the DUSP mRNAs examined in this study.**

All sequences are of human origin except for beta actin, which is from mouse.

| Gene name | Entrez ID | Gene symbol | Primer sequence | |
| --- | --- | --- | --- | --- |
| Dual Specificity Phosphatase 1 | 1843 | DUSP1 | FP | 5'-CTGCCTTGATCAACGTCTCA-3' |
|  |  |  | RP | 5'-ACCCTTCCTCCAGCATTCTT-3' |
| Dual Specificity Phosphatase 4 | 1846 | DUSP4 | FP | 5'-AGGCGGCTATGAGAGGTTTT-3' |
|  |  |  | RP | 5'-CACTGCCGAGGTAGAGGAAG-3' |
| Dual Specificity Phosphatase 6 | 1848 | DUSP6 | FP | 5'-ATGGTAGTCCGCTGTCCAAC-3' |
|  |  |  | RP | 5'-AATGGCCTCAGGGAAAAACT-3' |
| Dual Specificity Phosphatase 12 | 11266 | DUSP12 | FP | 5'-AGGCAATGGGATACGAAGTG-3' |
|  |  |  | RP | 5'-CCTTCACGGTGATCCAGAAT-3' |
| Dual Specificity Phosphatase 10 | 11221 | DUSP10 | FP | 5'-GCGGCAGTACTTTGAAGAGG-3' |
|  |  |  | RP | 5'-ATTGGTCGTTTGCCTTTGAC-3' |
| Dual Specificity Phosphatase 16 | 80824 | DUSP16 | FP | 5'-AGAAAATTTTGCCGTGGTTG-3' |
|  |  |  | RP | 5'-GAGTTGGCCCAGAAAATTGA-3' |
| Dual Specificity Phosphatase 2 | 1844 | DUSP2 | FP | 5'-TGTGGAGATCTTGCCCTACC-3' |
|  |  |  | RP | 5'-CTCCACCATCTGGTTGTCCT-3' |
| Dual Specificity Phosphatase 3 | 1845 | DUSP3 | FP | 5'-GACTCCGGCATCACATACCT-3' |
|  |  |  | RP | 5'-ATCTCACGGTTCTGCCTCAC-3' |
| Dual Specificity Phosphatase 5 | 1847 | DUSP5 | FP | 5'-GGAGGCCTTCGATTACATCA-3' |
|  |  |  | RP | 5'-AGGGCTCAGTGTCTGCAAAT-3' |
| Dual Specificity Phosphatase 7 | 1849 | DUSP7 | FP | 5'-CACTGGAGCCAGAACCTCTC-3' |
|  |  |  | RP | 5'-TAGGCGTCGTTGAGTGACAG-3' |
| Dual Specificity Phosphatase 8 | 1850 | DUSP8 | FP | 5'-CTGCTACCCATGAGCCTCTC-3' |
|  |  |  | RP | 5'-GGGCAGCAGTTTTTCACAGT-3' |
| Dual Specificity Phosphatase 9 | 1852 | DUSP9 | FP | 5'-GCATCCGCTACATCCTCAAT-3' |
|  |  |  | RP | 5'-ACAGTGACGGTGACAGAACG-3' |
| Dual Specificity Phosphatase 11 | 8446 | DUSP11 | FP | 5'-TCATGCAACCAGTCCACAAT-3' |
|  |  |  | RP | 5'-ATTGGGCTTCACATTCCAAG-3' |
| Dual Specificity Phosphatase 13 | 51207 | DUSP13 | FP | 5'-GCAGGGAAGTCTTCTTGCAG-3' |
|  |  |  | RP | 5'-CTCACACTGCTGCCGTAGAA-3' |
| Dual Specificity Phosphatase 14 | 11072 | DUSP14 | FP | 5'-TCCACAGTGTGAGCAGGAAG-3' |
|  |  |  | RP | 5'-GTCGACTTCCCAAAGAGCTG-3' |
| Dual Specificity Phosphatase 15 | 128853 | DUSP15 | FP | 5'-GGCAGCAGCTTGAAGAGTTT-3' |
|  |  |  | RP | 5'-CTCGGAGGCTGCTGAGTG-3' |
| Dual Specificity Phosphatase 18 | 150290 | DUSP18 | FP | 5'-GTGTGCCTTCCCAGTTCAGT-3' |
|  |  |  | RP | 5'-CGTGAGTTAGGGGAGTCAGC-3' |
| Dual Specificity Phosphatase 19 | 142679 | DUSP19 | FP | 5'-ACCAGGGTGACAACGCTAAC-3' |
|  |  |  | RP | 5'-TTGTGACCCTAGGAGCAACC-3' |
| Dual Specificity Phosphatase 21 | 63904 | DUSP21 | FP | 5'-TCATCATCTCAGGGTGTCCA-3' |
|  |  |  | RP | 5'-ATCAGCAATGGGGTCAAAAA-3' |
| Dual Specificity Phosphatase 22 | 56940 | DUSP22 | FP | 5'-AGCGGATTCACCATCTCAAA-3' |
|  |  |  | RP | 5'-CCTGGAGCTGTCTCTGGAAG-3' |
| Dual Specificity Phosphatase 23 | 54935 | DUSP23 | FP | 5'-AACTTCTCCTGGGTGCTTCC-3' |
|  |  |  | RP | 5'-GTCCACGATCTGCACGAAG-3' |
| Dual Specificity Phosphatase 26 | 78986 | DUSP26 | FP | 5'-GGAGACCAGGACATGGCTAA-3' |
|  |  |  | RP | 5'-ACAATGCACCAGGATCTTCC-3' |
| Dual Specificity Phosphatase 27 | 92235 | DUSP27 | FP | 5'-AAAGGGAAGAGTCCCCAGAA-3' |
|  |  |  | RP | 5'-CTCCCTGTCAGTGCTTCTCC-3' |
| Dual Specificity Phosphatase 28 | 285193 | DUSP28 | FP | 5'-GCCTGCCTAGTCTACTGCAA-3' |
|  |  |  | RP | 5'-CTGGAGCTGAGACCAGAAGC-3' |
| Glyceraldehyde-3-phosphate  dehydrogenase | 2597 | GAPDH | FP | 5'-TTCTTTTGCGTCGCCAGCCGA-3' |
|  |  |  | RP | 5'-GTGACCAGGCGCCCAATACGA-3' |
| Actin beta | 11461 | Actinb | FP | 5'-CTAAGGCCAACCGTGAAAAG-3' |
|  |  |  | RP | 5'-GGGGTGTTGAAGGTCTCAAA-3' |
